# Efficient Photoacoustic Image Synthesis with Deep Learning

**DOI:** 10.3390/s23167085

**Published:** 2023-08-10

**Authors:** Tom Rix, Kris K. Dreher, Jan-Hinrich Nölke, Melanie Schellenberg, Minu D. Tizabi, Alexander Seitel, Lena Maier-Hein

**Affiliations:** 1 Division of Intelligent Medical Systems, German Cancer Research Center (DKFZ), Im Neuenheimer Feld 223, 69120 Heidelberg, Germany; 2 Faculty of Mathematics and Computer Sciences, Heidelberg University, 69120 Heidelberg, Germany; 3 Faculty of Physics and Astronomy, Heidelberg University, 69120 Heidelberg, Germany; 4 HIDSS4Health—Helmholtz Information and Data Science School for Health, 69120 Heidelberg, Germany; 5 National Center for Tumor Diseases (NCT), NCT Heidelberg, a partnership between DKFZ and University Medical Center Heidelberg, 69120 Heidelberg, Germany; 6 Medical Faculty, Heidelberg University, 69120 Heidelberg, Germany

**Keywords:** photoacoustic imaging, deep learning, image synthesis, multispectral functional imaging, surrogate model, Monte Carlo simulation, Fourier Neural Operator

## Abstract

Photoacoustic imaging potentially allows for the real-time visualization of functional human tissue parameters such as oxygenation but is subject to a challenging underlying quantification problem. While in silico studies have revealed the great potential of deep learning (DL) methodology in solving this problem, the inherent lack of an efficient gold standard method for model training and validation remains a grand challenge. This work investigates whether DL can be leveraged to accurately and efficiently simulate photon propagation in biological tissue, enabling photoacoustic image synthesis. Our approach is based on estimating the initial pressure distribution of the photoacoustic waves from the underlying optical properties using a back-propagatable neural network trained on synthetic data. In proof-of-concept studies, we validated the performance of two complementary neural network architectures, namely a conventional U-Net-like model and a Fourier Neural Operator (FNO) network. Our in silico validation on multispectral human forearm images shows that DL methods can speed up image generation by a factor of 100 when compared to Monte Carlo simulations with $5\times 10^{8}$ photons. While the FNO is slightly more accurate than the U-Net, when compared to Monte Carlo simulations performed with a reduced number of photons ($5\times 10^{6}$), both neural network architectures achieve equivalent accuracy. In contrast to Monte Carlo simulations, the proposed DL models can be used as inherently differentiable surrogate models in the photoacoustic image synthesis pipeline, allowing for back-propagation of the synthesis error and gradient-based optimization over the entire pipeline. Due to their efficiency, they have the potential to enable large-scale training data generation that can expedite the clinical application of photoacoustic imaging.

## 1. Introduction

Multispectral photoacoustic imaging (PAI) is a novel spectral imaging technique that has shown great potential in a number of clinical disciplines: Pilot studies have demonstrated that it can be used to detect cancer and metastases [1,2,3,4,5,6], classify cancerous tissue [7], monitor temperature during tumor ablation [8], and diagnose and stage diseases such as inflammatory arthritis [9], psoriasis [10], and Crohn’s disease [11]. Most of these applications are based on the estimation of clinically relevant parameters such as tissue oxygen saturation in a spatially resolved manner.

PAI combines the advantages of optical and acoustic imaging by leveraging the photoacoustic effect, which provides high optical contrast based on the absorption of photons by chromophores in tissue as well as high spatial resolution up to several centimeters deep into tissue [12]. Performing PAI multispectrally potentially enables functional imaging of tissue properties. To this end, the determined absorption properties can be spectrally unmixed to obtain concentration maps of chromophores. This quantification is, however, very challenging as the estimation of the absorption from measured signals is an ill-posed problem [13].

**Figure 1:**
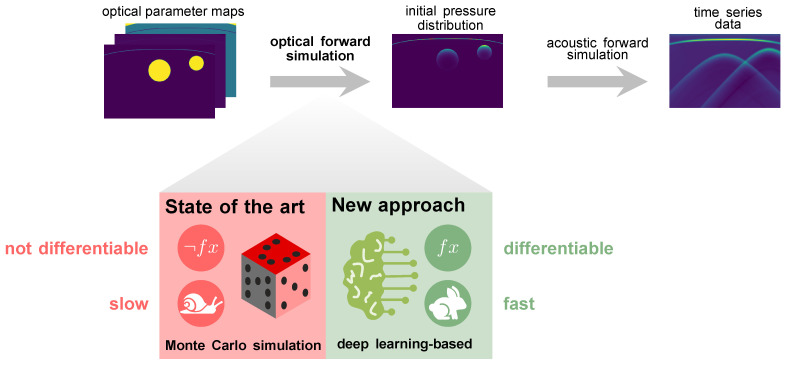
Our deep learning-based approach to simulating photon transport in biological tissue replaces slow state-of-the-art Monte Carlo simulations in the photoacoustic image synthesis pipeline.

So far, model-based quantification methods have not shown the required robustness in clinical settings [14]. Data-driven approaches, on the other hand, are highly promising [15,16] but suffer from a lack of reference methods for obtaining ground truth values of optical parameters in vivo for supervised learning settings. This issue is currently mitigated by simulating the entire photoacoustic image generation pipeline, as depicted in Figure 1. However, current state-of-the-art methods for simulating photon propagation in tissue are based on Monte Carlo simulations and are thus computationally expensive. Furthermore, these stochastic models are mathematically not inherently differentiable and can thus not be used for gradient-based optimization to improve the realism of the simulated images [17].

The underlying hypothesis of this work is that deep neural networks are capable of learning photon propagation patterns in biological tissue, thus enabling the efficient synthesis of photoacoustic images in a backpropagatable pipeline (Figure 1).

While there are similar approaches in other research areas [18,19], the only published deep learning-based approach in the field of photoacoustics that we are aware of is that of Bench and Cox in the context of creating an Ambient-GAN [20]. While the authors demonstrated the potential of deep learning to learn photon transport in tissue, a detailed analysis of the efficiency and accuracy of the optical forward surrogate model is missing. To close the present gap in the literature on this topic, this work focuses on two research questions:

1. How accurately can a deep learning-based model replicate initial pressure distributions produced with Monte Carlo simulations based on optical parameter maps of absorption and scattering coefficient as well as scattering anisotropy?
2. How large is the gain in run time of using deep neural networks compared to representative state-of-the-art Monte Carlo eXtreme (MCX) [21] simulations?

**Figure 2:**
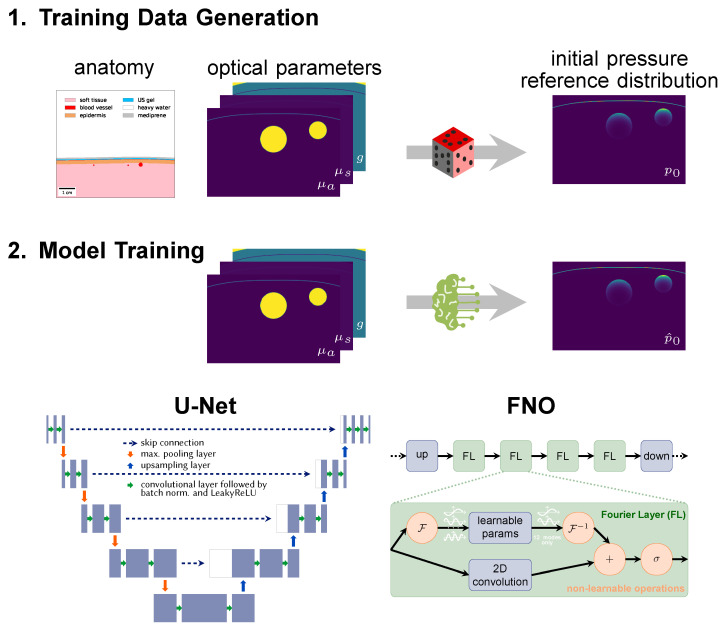
Neural networks are trained on synthetic data. (1) Image synthesis is based on a literature-based anatomy model (here: of the human forearm) and comprises the steps of tissue geometry generation, optical parameter assignment (absorption coefficient $\mu_{a}$, scattering coefficient $\mu_{s}$ and scattering anisotropy *g*), and simulation of the initial pressure distribution $p_{0}$. (2) Neural networks are trained with pairs of initial pressure distribution and corresponding parameter maps, here with either a modified U-Net with three input channels or a Fourier Neural Operator (FNO) network.

To this end, we explore two complementary neural network architectures, namely a U-Net [22] and a Fourier Neural Operator (FNO) [23] (Figure 2).

## 2. Materials and Methods

This work investigates if new deep learning-based approaches can be used instead of state-of-the-art Monte Carlo simulations for the optical forward simulation. To this end, two complementary model architectures are examined. One employs convolutional layers on multiple spatial scales as in image-to-image learning tasks, while the other one operates in Fourier space to capture the global context and thus approximates the underlying physical function. Section 2.1 describes the training data generation, followed by the model training in Section 2.2 and the experimental design in Section 2.3.

### 2.1. Training Data

A reference photoacoustic data set of human forearms was constructed by creating virtual tissues based on anatomical knowledge from the literature with the toolkit for Simulation and Image Processing for Photonics and Acoustics (SIMPA) [24]. As reported in more detail in [25], radial and ulnar arteries with accompanying veins were embedded in soft background tissue covered by skin (cf. Figure 2). Vessel locations and diameters were determined by randomly choosing from statistical distributions based on prior knowledge from the literature. Each tissue structure was assigned optical parameters (absorption coefficient $\mu_{a}$, scattering coefficient $\mu_{s}$, and scattering anisotropy *g*) based on values from literature represented in SIMPA’s tissue library [24,26]. Simulations were performed on 16 wavelengths in the visible and near-infrared spectral range, specifically from 700 nm to 850 nm in steps of 10 nm. A digital device twin of the MSOT Acuity Echo device (iThera Medical, Munich, Germany) including a 30 mm wide slit illumination was implemented in the SIMPA toolkit [24] (Find all specification details in the implementation of the digital device twin [27]) and used for modeling the illumination source. The highly parallelized and Graphics Processing Unit (GPU)-optimized software was used to simulate the photon propagation in tissue. Although the simulation results are very accurate when facilitating 5 × 10^8^ photons [28,29], the stochastic simulation process is computationally costly. Note that currently MCX only uses a single value for the scattering anisotropy instead of two-dimensional parameter maps. To account for this, the scattering coefficient was adjusted accordingly using the reduced scattering coefficient so that it is accurate in the diffuse regime but might be inaccurate in the quasi-ballistic regime.

The size of the two-dimensional optical parameter maps and resulting initial pressure distributions is 128 × 256 pixels, although the simulation is performed in three dimensions. With a pixel spacing of 0.15625 mm, an image covers a lateral length of 40 mm and depth of 20 mm. The entire synthetic data set consists of 1100 samples with 16 wavelengths each. The data were split via stratified sampling into a training (770 samples), validation (110), and test set (220) so that the models could learn from 14,080 image pairs during training and validation.

### 2.2. Deep Learning Models

The deep learning models were trained in a supervised manner with optical parameter maps of $\mu_{a}$, $\mu_{s}$, and *g* as input and estimations of the initial pressure distribution $p_{0}$ as output. Note that the wavelength was not explicitly fed into the neural networks but implicitly encoded in the optical parameter values. As the initial pressure distributions have large value ranges, they were logarithmically transformed before being used as estimation targets for the neural network according to the formula $p_{0}^{\prime }=\log_{10}\left(1+p_{0}\right)$ and retransformed afterwards.

Two different neural network architectures were used. (1) The first network is based on the well-known U-Net [22], which is commonly used in image-to-image learning tasks in the biomedical computer vision domain: As shown in Figure 2, the U-Net is a convolutional neural network which works on multiple scales. Residual skip connections enable the U-Net to combine long-distance features with details and thus have a large receptive field. Compared to the original U-Net, the architecture was slightly modified to consist of three input channels and only a single output channel. A depth of 4 was used, i.e., the input was downsampled four times with max-pooling layers. Dropout layers with 0.5 probability were implemented to regularize the learning and batch normalization layers, and LeakyReLU activations were applied. (2) As a second network, we used an FNO network (Li et al. [23]), which first projects the data up into a 128-dimensional latent space with a linear layer. This step is followed by the traversal of four Fourier layers with two paths each. In one path, spectral convolutions are performed, i.e., instead of normal convolutional operations, the data is Fourier-transformed and multiplied with learnable parameters. As a means of regularization, only the 12 lower modes are used, while the higher ones are clipped. This helps the network to focus on learning the low frequencies. In the other path, a two-dimensional convolution is performed which supports learning high-frequency features. Ultimately, the data passed through both paths are concatenated and fed into a Gaussian Error Linear Unit (GELU) activation function [30]. The usage of FNO networks was motivated by the solution of partial differential equations in Fourier space. The FNO architecture has already been applied in the estimation of photoacoustic wave propagation [31] and other physical phenomena such as fluid dynamics [23]. In contrast to convolutional neural networks, which capture local features such as edges and shapes in their kernels and only learn the global context by pooling or down-sampling, the Fourier layers can learn global sinusoidal features. They are therefore better suited for representing continuous functions [23] such as the Radiative Transfer Equation, which is the underlying equation for the photon propagation.

Both models were implemented with PyTorch and trained for 200 epochs with a batch size of 16, an initial learning rate of 0.01, and a reduce-on-plateau learning rate scheduler that halves the learning rate after a patience of 5 epochs and a threshold of 1 × 10^−4^. The best models were chosen based on the minimal validation loss per epoch, which was achieved in epoch 73 for the U-Net and 41 for the FNO, respectively. While the U-Net uses a mean squared error loss, the FNO is optimized according to the slightly different $L_{2}$ loss. Both models were optimized with the Adam optimizer [32] and a weight decay of 1 × 10^−4^. As a data augmentation method, the input image pairs were randomly flipped left and right.

### 2.3. Experimental Design

To validate the models’ estimation performance, several experiments were conducted on the held-out test set. For assessment of the estimation accuracy, the mean absolute error (MAE), structural similarity index measure (SSIM) [33], and peak signal-to-noise ratio (PSNR) were computed between the estimated initial pressure distribution images and their corresponding reference. As samples feature varying signal intensity ranges, they were max-normalized by dividing the estimations and reference initial pressure distributions by the maximum pixel value of the reference image.

To determine the accuracy of the deep learning-based models in comparison to the Monte Carlo baseline, the same samples were simulated with a reduced number of photons. Once the error rate of the deep learning models equals that of the simulations with reduced photon count, intuition can be derived on how much the deep learning estimations deviate from the reference Monte Carlo simulations.

A fair comparison between the run time of the deep learning models and Monte Carlo simulations is challenging, as the Monte Carlo run time not only depends on the image resolution but also on the photon count. We took this into account by choosing a representative simulation setting with a resolution and photon count corresponding to that of regular use cases [20,34]. Furthermore, all computations are inherently hardware-dependent, so run times of multiple samples and using all 16 wavelengths were determined. All computations in our work were performed on a Ubuntu 22.04.2 LTS with NVIDIA Corporation GA102 [GeForce RTX 3090] GPU and AMD^®^ Ryzen 9 5900x 12-core processors × 24.

## 3. Results

**Figure 3:**
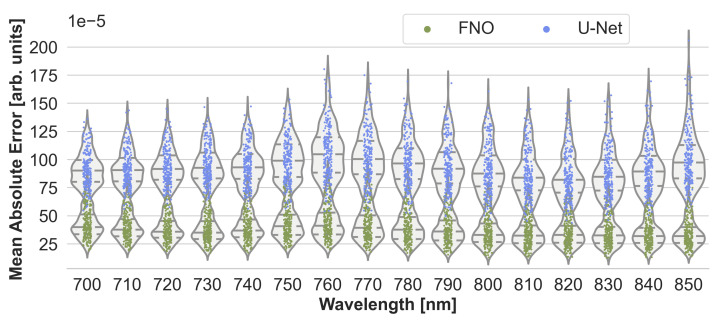
Performance of the Fourier Neural Operator (FNO) and the U-Net when using the Monte Carlo eXtreme (MCX) reference initial pressure distributions. According to the sample-wise max-normalized mean absolute error (MAE), the FNO consistently slightly outperforms the U-Net on the 220 test cases.

**Figure A1:**
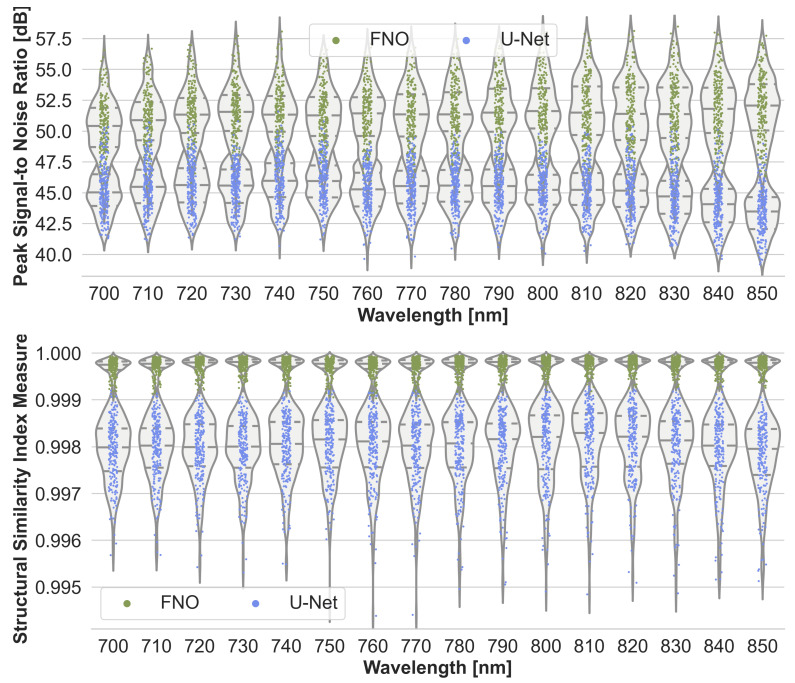
Performance of the Fourier Neural Operator (FNO) and the U-Net when using the Monte Carlo eXtreme (MCX) reference initial pressure distributions. According to the sample-wise max-normalized peak signal-to-noise ratio (**top**) and structural similarity index measure (**bottom**), the FNO consistently slightly outperforms the U-Net on the 220 test cases.

To assess the accuracy of the neural networks, their estimation performance on the held-out test set consisting of 220 samples was plotted as shown in Figure 3. For each sample, the estimated initial pressure distribution image from each wavelength was compared with the reference image from the MCX simulation. Figure 3 presents the max-normalized MAE of the FNO network and the U-Net model, and Figure A1 in the appendix the peak signal-to-noise ratio and structural similarity index measure, respectively. While the FNO slightly outperforms the U-Net, the error rates for each model are mostly similar across the wavelengths. Therefore, only results from one wavelength at 800 nm will subsequently be presented.

**Figure 4:**
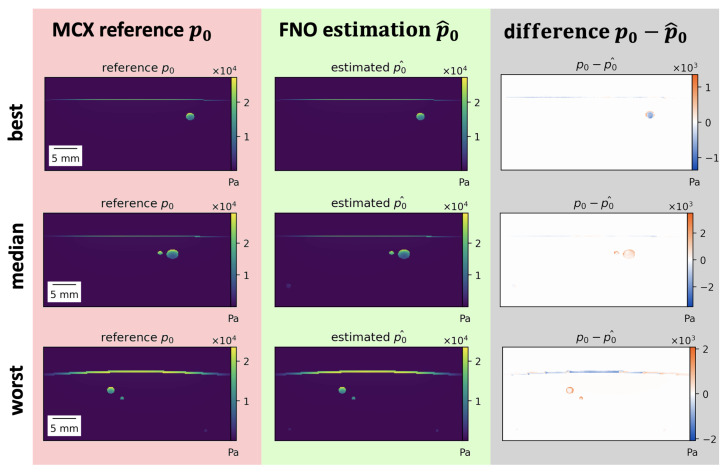
Results of initial pressure distribution $p_{0}$ estimation for the Fourier Neural Operator (FNO) $\hat{p_{0}}$. Best, median, and worst (**top** to **bottom**) performing samples for the FNO according to the normalized mean absolute error (MAE) on 800 nm. Most misestimations occur in high-absorbing structures such as vessels or skin. Poorly performing samples mostly feature thicker skin or high-absorbing vessels close to the skin.

Estimations of the best, median, and worst performing samples on 800 nm are shown in Figure 4 for the FNO. While the estimations closely resemble the reference, misestimations can be detected in high-absorbing structures such as vessels and skin. Similar results were obtained for the U-Net.

**Figure 5:**
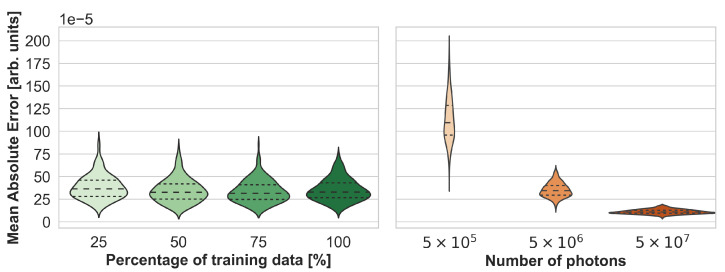
Synthesis accuracy measured with the max-normalized mean absolute error (MAE) as a function of the number of training cases for Fourier Neural Operator (FNO) network (**left**) and the number of photons for Monte Carlo eXtreme (MCX) simulations (**right**). Note that the reference simulations, which were used to compute the mean absolute error (MAE), were performed with 5 × 10^8^ photons.

**Figure 6:**
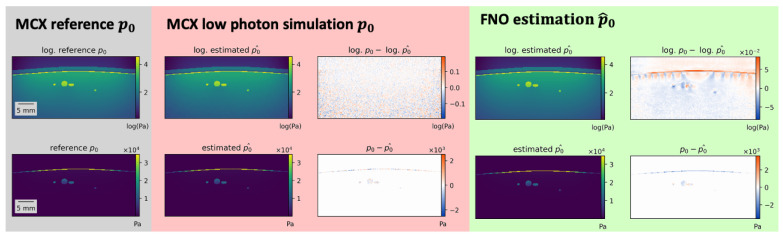
Estimation errors of Fourier Neural Operator (FNO) network and simulation errors of Monte Carlo eXtreme (MCX) of the same sample with fewer photons are different in nature. While deep learning estimations show errors at anatomical structure boundaries, MCX simulations with fewer photons contain general unstructured noise (apparent in the logarithmic visualization in the top row).

Using more training data only slightly improved the performance before reaching saturation, as depicted in Figure 5. In this experiment, the FNO was trained on 25%, 50%, and 75% of the training samples. Figure 5 also shows that the MAE on the test set is similar to that of MCX simulations performed with 5 × 10^6^ photons instead of the reference simulations which used 5 × 10^8^ photons. The type of error, however, is different, as shown in Figure 6. While Monte Carlo simulations with fewer photons generally result in noisier images, especially with increasing depth, the errors of neural network estimations occur at structure boundaries.

**Figure 7:**
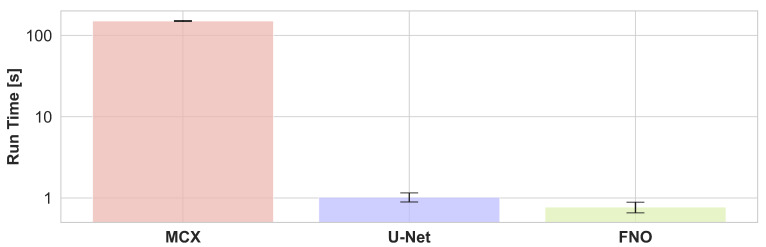
During inference, U-Net, and Fourier Neural Operator (FNO) networks estimate the initial pressure distribution two orders of magnitude faster than Monte Carlo simulations from Monte Carlo eXtreme (MCX) with representative configurations. Results were obtained by averaging the run time of the optical forward step of 22 samples and all 16 wavelengths, i.e., the reported run time is for one wavelength of a single sample. The error bars indicate the standard deviation.

The run times of a photon propagation simulation for one wavelength averaged over randomly chosen 10% of the test set and all 16 wavelengths with MCX and estimations of the U-Net and FNO are presented in Figure 7. It can be seen that the inference of neural networks is two orders of magnitude faster, about or even under a second compared to about two minutes for the MCX simulation. The FNO is slightly faster than the U-Net.

## 4. Discussion

Deep learning methodology is already used in the field of photoacoustic imaging for tasks such as the optical and acoustic inverse problem [15,35], image post-processing [36], and structure segmentation [37] as discussed in recent reviews [38,39,40]. While the lack of labeled in vivo data often constitutes a major obstacle, the speed-up achieved through deep learning methods and their unique capability of solving complex problems are substantial advantages. With this contribution, we are, to the best of our knowledge, the first to assess the feasibility of utilizing deep neural networks for simulating the photon propagation in tissue for PAI and to present a back-propagatable, fast, and accurate method for synthesizing realistic photoacoustic images. In particular, we investigated a U-Net-like model as well as an FNO network and compared their simulation results to state-of-the-art Monte Carlo simulations [13,41]. Although very accurate when choosing a configuration with enough photons and high resolution, these simulations are computationally very expensive when generating the large amounts of synthetic data needed to train quantification networks. This issue can be solved by using neural network surrogate models. We could show that both learning-based approaches yield error rates comparable to Monte Carlo simulations created with a hundred times fewer photons while achieving run times faster by two orders of magnitude. In contrast to Monte Carlo simulations, the employed learning-based approaches can be used as inherently differentiable surrogate models in the photoacoustic image synthesis pipeline, allowing for back-propagating the synthesis error and performing gradient-based optimization over the entire pipeline. Thus, the realism of the synthetic images can be improved by also optimizing the generative volume creation models as well as the initially literature-based optical parameter values through learning from in vivo data.

Compared to the state-of-the-art Monte Carlo approach, the nature of the error experienced using learning-based methods is different. While for Monte Carlo simulations, the error is dominated by general stochastic noise, misestimations at structure boundaries are prominent for the neural network approaches. Both models often struggle with high-absorbing and non-homogeneous tissue structures, such as vessels and skin. This might be due to the fact that these structures present higher intensity values and more complex shapes compared to homogeneous background tissue. In particular, samples with thicker or deformed skin and irregular vessels exhibit larger errors. This behavior might be unfavorable in a clinical context as most applications target the vessels or skin as structures of interest. However, as the error rate is similar to that of Monte Carlo simulations with two orders of magnitude fewer photons, the results suggest that the accuracy of our data-driven approaches is high enough for synthesizing useful photoacoustic images.

Our study further examined the computational efficiency of the neural networks via their inference run time. The results show that the FNO and U-Net estimations outperform the MCX simulations by two orders of magnitude. Note that all computations inherently rely on the hardware they were performed on, so small deviations might occur on other hardware. However, the inference run time only constitutes one aspect of efficiency considerations. Training neural networks requires time and resources. Supervised deep learning in particular requires training data, which in this case also had to be generated with computationally expensive Monte Carlo simulations, thus adding to the computational cost besides neural network inference. It is thus expected that once the training data is generated and models are trained, neural network surrogate models can be employed much faster. Note that there are several ways to speed up Monte Carlo simulations, e.g., by using fewer photons or a lower resolution. To ensure a fair comparison to the neural networks, however, we chose to pick a representative simulation configuration with a photon count and resolution that is commonly used in PAI simulations and the highly parallelized and run time optimized MCX simulation tool. Further note that the reference MCX simulations were performed in three dimensions to provide accurate simulations, especially regarding out-of-plane light-tissue interactions, and the two-dimensional center slice was cropped out at the end of the simulation pipeline. The neural networks, however, operated only on two-dimensional images, which additionally reduced their run time.

Our results show that the FNO is slightly faster than the U-Net, which can be explained by the smaller number of parameters in the FNO. In general, fast neural network surrogate models allow synthesizing photoacoustic images at a speed sufficient for generating large enough data sets for the data-driven quantification of photoacoustic images.

It is worth noting that to apply deep learning-based surrogate models to new data sets with other properties or device configurations, they need to be trained first. As currently no generic model for different body sites, optical parameter ranges, or device geometries exists, computationally expensive retraining and generation of data might still be necessary, thus limiting the applicability and efficiency of this approach in comparison to physics-based Monte Carlo simulations. This issue could, however, be circumvented by the sharing of trained models. It further highlights the need for more research to be conducted before generic optical forward surrogate models can be obtained.

It must be noted that in our experiment, the wavelength was only implicitly used as input during the training via the optical parameters. Despite this, the resulting estimations were good for the entire range of used wavelengths. In the future, it would be interesting to investigate the extrapolation case of estimations outside the training wavelengths and the interpolation between wavelengths. A further limitation of our experiment is that it was only performed using one photoacoustic device geometry and solely data from human forearms, as opposed to a wider variety of body regions. Thus, all samples were in-distribution. Future research should thus be dedicated to investigating the out-of-distribution case.

Another limitation of surrogate models is that they will most likely not be more accurate than the reference methods they were trained on. Thus, the quality of the MCX simulation limits the models’ accuracy. To mitigate this limitation, the neural network estimations can, however, be further optimized along the entire photoacoustic image synthesis pipeline, resulting in higher realism, also in the optical forward step. In general, a trade-off between synthesis speed and accuracy needs to be defined when training deep learning surrogate models.

An alternative approach for fast photon propagation models might be performing Monte Carlo simulations with lower resolution or fewer photons and, respectively, upsample or denoise afterwards. Ardakani et al. presented a deep neural network that denoises low photon simulations to achieve more accurate, fast simulations [42]. Their model is, however, not differentiable and thus not suitable for gradient-based learning-to-simulate approaches for photoacoustic images. Other alternative solutions include numerical methods such as finite elements; however, these are only feasible in small and simple as opposed to more realistic simulation scenarios [13,41]. Therefore, an inherently differentiable deep learning-based approach such as the one proposed further exemplifies the benefits of using deep learning in photoacoustic image synthesis, offering improvements in both accuracy and efficiency.

Future work should address the validation of our deep learning-based photoacoustic image synthesis approach by using the synthesis results in a downstream task to demonstrate equivalence to the state of the art. To this end, the back-propagation capability of the neural networks might also be used to show a benefit over non-differentiable models. Although the FNO network is internally already physics-related by using spectral convolutions, including more prior knowledge from physics not only in the neural network architecture but also in the entire deep learning approach might be beneficial for achieving even more accurate results and faster model convergence.

In conclusion, this proof-of-concept study demonstrates that the simulation of photon propagation in biological tissue can be efficiently and accurately accomplished using deep learning methods. Being an essential and inherently differentiable part of photoacoustic image synthesis, this enables fast simulation of large amounts of photoacoustic data, which paves the way for training models capable of recovering functional tissue properties.

## Data Availability

The in silico human forearm data set and trained models weights are provided on request.

## Abbreviations

The following abbreviations are used in this manuscript:

FNO	Fourier Neural Operator
GPU	Graphics Processing Unit
MCX	Monte Carlo eXtreme
PAI	photoacoustic imaging
MAE	mean absolute error
SIMPA	toolkit for Simulation and Image Processing for Photonics and Acoustics
